# Supplementary material for: Towards a cybersecure and privacy enhanced smart grid: A blockchain enabled federated learning framework
Source: PLoS One. 2026 Mar 2;21(3):e0342454. doi: 10.1371/journal.pone.0342454 (PMC12952658; doi:10.1371/journal.pone.0342454)
Supplement: Attachment — Submitted filename: Response to Reviewers.pdf [file pone.0342454.s001.pdf]

Editor Comments

We thank the Editor for this crucial observation. Per the specific instructions, we have prioritized the "readability" and "flow" of the presentation. We have also refined the language and structure to close the gaps in the presentation. We believe the manuscript is now much more readable and cohesive.

We have also incorporated all of the changes suggested by the reviewers in detail as provided below.

Reviewer #1

Reviewer #1: This paper proposes a secure and privacy-preserving framework that integrates a dual layer blockchain architecture, federated learning (FL) and central differential privacy (DP) to address challenges related to user privacy and system-level security holistically.

Concern # 1: In the abstract, you need to mention the research gaps in the current techniques for addressing these user privacy and system-level security challenges. This should be done prior to introducing the proposed framework.

Authors Response: We thank the reviewer for their valuable feedback. To resolve this concern, we have updated the manuscript to include the research gaps in current techniques for addressing user privacy and system level security issues. The updated abstract is as follows:

In smart grids, data collection is carried out through smart meters and devices of the Internet of Things, which are installed in the home, allowing to predict the demand for electricity and optimize the distribution of energy. Although the smart grids improve efficiency of operations for end users, they simultaneously present pronounced challenges regarding user privacy and security at the system level. In the context of conventional centralized machine learning, paradigms risk breaching the raw data of consumers, while decentralized paradigms often lack strong mechanisms for verifying identity or ensuring traceability. Existing federated learning systems often lack client level differential privacy, secure aggregation, and decentralized identity protection, leaving them vulnerable to privacy leakage and inference attacks. Blockchain based solutions typically expose model updates or use single layer identifiers. This paper introduces a secure and privacy preserving architecture that combines a dual layer blockchain architecture,

federated learning (FL) and central differential privacy (DP) to thoroughly solve these challenges. The proposed system includes a dual layer blockchain system that ensures secure and tamper resistant logging of client interactions and protects client identities by storing salted cryptographic hashes. This design provides both traceability and anonymity, and thus maintains the integrity of participation while obfuscating sensitive identifiers. Privacy is guaranteed by storing raw data in client devices and sending only model updates for central aggregation. At the server side, Gaussian noise is added to the aggregated model parameters to achieve central DP, so as to reduce the risks of inference attacks on user data. Implementation of the proposed framework was performed based on Flower to test the PRECON (Pakistan Residential Electricity CONsumption) dataset, which consists of real-world household electricity consumption data. Multiple machine learning models were benchmarked and out of all the models, Random Forest performed best with the performance metrics of Mean Absolute Error (MAE) of 0.153, Mean Absolute Percentage Error (MAPE) of 0.085 and Mean Squared Error (MSE) of 0.143. The results showed that the proposed framework improved data privacy, preserved the forecasting accuracy and security in smart grid environments.

Concern # 2: To mitigate against reader confusion, all acronyms must be written in full the first time they appear within text. For instance, you have used ' MAE, MAPE, MSE' in the abstract devoid of stating what they stand for in full.

Authors Response: We thank the reviewer for the valuable feedback. We have reviewed the document and fixed all the acronyms, especially in the abstract as seen below:

Implementation of the proposed framework was performed based on Flower to test the PRECON (Pakistan Residential Electricity CONsumption) dataset, which consists of real-world household electricity consumption data. Multiple machine learning models were benchmarked and out of all the models, Random Forest performed best with the performance metrics of Mean Absolute Error (MAE) of 0.153, Mean Absolute Percentage Error (MAPE) of 0.085 and Mean Squared Error (MSE) of 0.143.

We have also included a table below the Introduction section that list all the acronyms present in the document. As shown below:

Table 1. List of Acronyms

Acronyms	Description
AI	Artificial Intelligence
BeFL	Blockchain enabled Federated Learning framework
B-FRL	Blockchain-Federated Reinforcement Learning
CCPA	California Consumer Privacy Act
CID	Client ID
DP	Differential Privacy
FL	Federated Learning
GAN	Generative Adversarial Network
GDPR	General Data Protection Regulation
HGB	HistGradient Boosting
HIPAA	Health Insurance Portability and Accountability Act
HN	House Number
IID	Independent and Identically Distributed
IoT	Internet of Things
KNN	K-Nearest Neighbor
LR	Linear Regression
MAE	Mean Absolute Error
MAPE	Mean Absolute Percentage Error
ML	Machine Learning
MLP	Multi-Layer Perceptron
MSE	Mean Squared Error
PAFLM	Privacy-Preserving Asynchronous FL Mechanism
PRECON	Pakistan Residential Electricity CONsumption
QFEVAL	Quantum Federated Ensembled Variational Adaptive Learning
R^2	Coefficient of Determination
RF	Random Forest
RPC	Remote Procedure Call
SG	Smart Grid
SVR	Support Vector Regression
UUID	Universally Unique Identifier

Concern # 3: What is the need for 'Author summary' section?

Authors Response: We thank the reviewers for pointing out this mistake. We have removed this section in its entirety.

Concern # 4: The Introduction is not elaborate enough so as to bring out the research area and problem domain. Therefore, you need to expand this section so that you adequately describe the research area and problem domain.

Authors Response: We thank the reviewer for their valuable feedback. We have reviewed the Introduction section and expanded the section to include more discussion on the research area as well as the problem domain. The updated section is provided below:

In the past couple of years, the AI industry has flourished so much that we have shifted from mechanical devices to smart machines. Grids are adapted into Smart grids with smart meters and edge devices to control and manage resources in smart cities. Smart grid (SG) [1] is a radical concept introduced to collaborate power systems' production, distribution, and generation under one umbrella. The aim of SG is to efficiently manage the delivery of energy to the end-users from different generation sources while making use of advanced Artificial Intelligence (AI) algorithms for monitoring of power consumption as well as generation in real time [2, 3]. Real time data acquisition in smart grids (SG) is carried out by smart meters, sensors and Internet of Things (IoT) devices, and therefore enables accurate load forecasting and demand management along with energy distribution optimization. These systems improve operational efficiency, integration of renewable energy resources and implementing dynamic demand response strategies. However, these benefits bring significant risks related to the data confidentiality, privacy of consumers and overall security of the grid infrastructure. In SGs real time data is gathered using smart meters, sensors, and IoT devices to enable accurate load forecasting, efficient management of demand and optimization of energy distribution [4, 5]. These systems play a role in improving operational efficiency, facilitating the integration of renewable energy resources and implementation of dynamic demand response strategies but simultaneously it raises significant concerns regarding data confidentiality, consumer privacy, and grid infrastructure security [6, 7].

In SG architectures, raw household data are aggregated and sent to centralized servers to be used to train machine learning (ML) models for load forecasting, renewable energy integration and consumption analysis. While centralization is a way to improve the performance of models and to enable the analysis of large data sets, it simultaneously exposes the sensitive data of consumers to potential misuse and privacy violations, data breaches, non-compliance, and cyber attacks [8, 9]. Furthermore, the absence of transparent mechanisms for verifying the provenance of data and ensuring input integrity makes these systems susceptible to adversarial manipulation and tampering. In SG environments, the constant exchange of information between smart meters,

edge devices, and central servers' results in the exposure of confidential household information, including device usage patterns and meter IDs, to potential tampering, spoofing or unauthorized access [10, 11]. Existing communication and logging mechanisms are not sufficient to ensure integrity, hinder manipulation or validate the authenticity of the participating entities.

So, to address this issue, machine learning's approach Federated Learning [17] is a viable solution which consists of edge-enabled AI technologies. FL is a distributed ML technique that allows multiple systems to collaboratively train a global model without sharing, with a central server, any type of data. In the context of SG, FL can be used to improve energy management, load forecasting, and renewable energy forecasting while maintaining the privacy as well as security of data and reducing communication overhead. The application of blockchain technology would also help to increase the security, transparency and auditability of the consumer's sensitive data. The proposed Blockchain enabled Federated Learning framework (BeFL) ensures data protection standards suggested by General Data Protection Regulation (GDPR) [12] and California Consumer Privacy Act (CCPA) [13] in regulation compliance, rigid data locality and pseudonymization by using SHA-256 hashing with salting and blockchain based auditability. No raw or identifiable information is transmitted or stored at the center, therefore making the system comply with contemporary privacy by design principles.

In this paper, we propose an integrated framework which combines FL, centralized DP, and a dual layer blockchain architecture to secure users' identities and guarantee that users can participate in collaborative learning. This paper presents a novel privacy and security aware framework that consists of federated learning with blockchain based aggregation for smart grid forecasting. The proposed system maintains data hiding by using a dual layer blockchain to log cryptographic hashes of client sensitive data for data security and applies gaussian noise during model aggregation to meet differential privacy standards [21][24]. By addressing the limitations of prior work, this framework provides a secure and privacy preserving solution for collaborative learning in smart grid environments.

The proposed framework is not restricted to SGs for forecasting, but it can be used in other sectors, where data privacy and trust are of the most importance. In the medical field it can enable model training across hospitals while adhering to data protection laws like Health Insurance Portability and Accountability Act (HIPAA) [14] and GDPR [12]. In smart cities, it

can be used for privacy aware mobility or environmental forecasting using edge IoT devices. In the financial sector, the framework can be applied to identify fraud or to optimize the prediction of risk without exposing client sensitive transactional data. Additionally, for the industrial IoT environments, it helps support predictive maintenance and process optimization across the distributed manufacturing units while protecting proprietary information. By maintaining data locality, input origin verifiability, and individual contribution protection, the framework provides a scalable, secure, and privacy-conscious decentralized AI solution for multi domain use.

Concern # 5: The 'Problem Statement' section is not well done as you have began by stating that 'These vulnerabilities can be addressed by incorporating a blockchain-based security layer that ensures tamper-proof storage, immutable audit trails, and secure identity protection through the use of salted cryptographic hashes.'

- It is not clear which vulnerabilities you are alluding to.
- This section is not written in a logical and concise manner.

Authors Response: We thank the reviewer for the valuable feedback. We have reviewed the Problem Statement section, and we have updated it accordingly. The updated manuscript is as follows:

In traditional smart grid architectures, raw data is normally obtained from different households which is then aggregated for application of various ML models [9] for forecasting such as load management and renewable energy forecasting. Though this approach of storing data in a central server is effective in terms of model performance. But at the same time these approaches risk the exposure of highly sensitive consumer data to potential cyberattacks, non-compliance or misuse [7][8]. Also, there are high risks of tampering and adversarial manipulation [10][11] due to the unavailability of any type of transparent mechanism for ensuring input integrity and verification of data origin. Overall, this centralized paradigm creates several vulnerabilities in terms of both security and privacy, some of which are also provided below:

- The transmission and storage of raw user data increase the risk of data breach and surveillance.
- Lack of data integrity checks, which makes systems vulnerable to tampering or injection attacks.

- Lack of decentralized validation of the origin of data or identity of participants, which increases lack of trust among participants collaborating to learn.
- Privacy of the individual is compromised during model training.

In order to counter such issues, recent research works have explored FL and blockchain as an alternative. Blockchain ensures the security of sensitive data while the concept of FL introduces a decentralized approach where clients train ML models locally with their own data and then forward only the model updates or parameters to the central server [15][18]. Through this approach, the exposure of raw data is significantly reduced and also the user privacy is maintained as the local data remains on edge devices. However, FL as a standalone method does not completely provide protection against privacy leakage as the shared model parameters may still be breached in order to obtain partial insights into the underlying data distribution [19][20]. In order to counter this, the technique of DP has been utilized by different research works. Through the use of DP, random noise is introduced into the model aggregation which overall limits the amount of information that could be inferred from individual model parameters [21][22]. As such, many existing frameworks apply DP in isolation, which can be crucial for maintaining trust and auditability in collaborative learning environments. However, conventional DP often introduces excessive Gaussian noise to ensure strong privacy, which compromise the accuracy of the training model especially when applied to small datasets. Overall, there is an urgent need of comprehensive framework with enhanced privacy and security measures to not only guarantee user data confidentiality but also to guarantee traceable and tamper resistant provenance of data and verify involvement in collaborative model training.

Concern # 6: The 'Key Contributions ' is not well done. Th main/key contributions must be stated in point form; with each of these points reflecting some kind of notable novelty.

Authors Response: We thank the reviewer for their valuable feedback. We have reviewed and updated the Contribution section accordingly. The updated manuscript is as follows:

In light of the aforementioned challenges, a privacy preserving framework is proposed that helps secure the personal information of power consumers. The proposed framework employs the use of FL, blockchain and central DP. The salient features of the proposed framework are discussed below:

- A dual layer blockchain structure is introduced, in which sensitive identifiers such as client ID (CID)and house numbers (HN) are anonymized using salted cryptographic hash functions before being submitted to smart contracts. This design means that no raw identifiers are ever exposed to the on-chain, significantly reducing the risk of re-identification.
- The framework also integrates duplicate detection under smart contracts to prevent redundant data submissions and increase the efficiency of the blockchain.
- This framework also considers the inclusion of FL to ensure privacy as consumer data is trained on federated clients only. The local parameters from this training are forwarded to a central server for model aggregation while keeping the raw data at client devices.
- To reinforce privacy even more, at the FL server side (on the model aggregation stage) a central differential privacy mechanism is employed through which Gaussian noise is added to the aggregated model parameters to prevent side channel information leakage but preserve the utility of the model.

Furthermore, a thorough analysis is carried out by taking into account some measurements such as gas usage, transaction cost, latency, hash collision rate, raw data leakage, and salt entropy. The results show that the proposed framework achieves robust privacy guarantees with low computational and transactional overhead and is therefore applicable to secure smart grid and IoT applications.

Concern # 7: In the 'Literature Work' section, you have failed to bring out the research gaps in each of the presented works. It is therefore extremely difficult to discern which gaps this study sought to bridge.

Authors Response: We thank the reviewer for their valuable comments. We have updated the manuscript to accommodate all of the research gaps present in current literature work. The updated Literature Work section is as follows:

Privacy and Security Preservation in Smart Grids

The distribution of data within the SG poses great challenges to the processing of large volumes of information. Centralized aggregation of data would place huge burdens on communication

bandwidth and computational resources [8]. Conventional strategies include task partitioning, parallel execution, and data sharing as well as the usage of industrial applications. Nevertheless, many methods are based on direct exchange of proprietary data between the participants, which is insecure and compromises privacy [7] [9][10]. The SG infrastructure aims to keep data confidential; hence it is essential for data to be secured in the analytics pipeline to avoid being exploited by malicious users. In 2016, Google introduced FL which is a new method for decentralized training. FL has since been used in a range of research fields and applications including mobile technology, healthcare and the internet of things [5][6].

A secure framework of FL and blockchain has been proposed by a group of researchers [11]. In light of cyber threats, as well as privacy issues, this framework expressly avoids transmission of raw local data. Each node, in the process of the training, updates the individual parameters of its model before broadcasting the refined models to a central authority or to fellow nodes within the network. Although the framework enhances stability and fairness in federated digital-twin modeling, it is limited by residual privacy risks, restricted support for out-of-distribution users, and the smoothing constraints of its LSTM architecture.} Lyu et al. [15] suggested a federation based deep learning approach that considers local credibility, fairness, and privacy protection. Privacy ensured with layered encryption techniques and private Generative Adversarial Networks (GANs) were used by nodes to assess input and performance. Nodes also collaborated to evaluate contribution impact. Gradients were encrypted during training to maintain confidentiality, leading to the higher model price. However the framework is evaluated in an IID environment which is limited by its reliance on an honest but curious threat model with insufficient robustness against poisoning or Byzantine adversaries. Zhai et al. [16] introduced a novel structure integrating link reliability for predicting power grid energy usage. The framework considered timing, client selection, and delay, but neglected privacy concerns during model training. Sharing regional data during FL posed risks from attackers. The framework has limited its applicability to realistic heterogeneous smart grid environments. Though the impact of non-IID data, large-scale deployments and real wireless dynamics on model accuracy remains unexplored in this paper. Taik et al. [17] proposed FL for smarter energy trading where an individual could predict energy demand and devise a trading plan accordingly.

Smart meter analysis displays potential as an evolving field of study. Security vulnerabilities such as poisoning or backdoor attacks are acknowledged but it has not been experimentally addressed and provided no comparison with state of the art FL baselines. Furthermore, the prosumer decision model and prediction horizons remain simplified, limiting the framework's applicability to large, heterogeneous smart grid deployments. In a publication, [18] researchers employed FL as a method to protect people's privacy for uses such as forecasting behind the meter solar photovoltaic power. With increasing cyberattacks and vulnerabilities in smart meters and distributed grid appliances, research on anomaly detection and theft prevention has also gained a lot of popularity. However this study was limited to only one dataset and one FL model, and does not address adversarial, scalability, or real device constraints, limiting its real world applicability. In research, Alshehri et al. [19] suggested a federated deep learning approach for detecting zero-day attacks over electricity meters. Moreover, Liu et al. [20] showed that decentralized federated learning frameworks can be resistant to data integrity and trojan insertion attacks. The limitations in both studies rely on simplified simulations with ideal communication, limited attack types and non-IID scalability validation, which limits their applicability to real world smart grid deployments.

Privacy and Security Preservation Approaches Using ML/FL

Most studies concentrate on utilizing encryption methods on the users' original data, which is quite challenging and requires significant effort to execute. This problem can be resolved by using FL which has been utilized in recent research to cohesively train and evaluate ML models without compromising the privacy of data stored on secure nodes. The only information that is transmitted to the server is the ML algorithms' specifications, and this approach has the potential to significantly minimize security and computational challenges associated with centralized ML [21-23]. In a paper [24] scholars suggested Privacy-Preserving Asynchronous FL Mechanism (PAFLM) for an edge network which could enable numerous edge nodes to perform more effective FL without disclosing their private data. The suggested technique, as compared to conventional distributed learning, compresses communication between nodes and the parameter server during training without sacrificing accuracy. Although the paper aims to reduce communication costs, it does not model realworld IoT network challenges, such as intermittent connectivity, packet loss, bandwidth fluctuation, or device dropout, even though IoT

environments are inherently unstable. A group of Chinese academics developed the PEFL protocol [25] , which protects the confidentiality of training data, both during and after the training process and even when numerous entities overlap with one another. The paper states PEFL works for large-scale scenarios, but no experiments are conducted with thousands of clients or high-dimensional industrial gradients. In a paper [26], by combining additively homomorphic encryption with differential privacy, researchers devised a protocol for an effective and privacy preserving federated deep learning system based on the stochastic gradient descent technique. This protocol would be able to stop data from being exploited by untrusted parties. However, the paper does not consider network delays, packet loss, dropout patterns, or scalability bottlenecks in real-world FL communication.

Machine learning and FL techniques have become influential in preserving data privacy during smart grid analysis [51]. The DP^{2}-NILM model introduced by Shao et al. [27] utilizes FL for non-intrusive load monitoring without exposing users' data. Similarly, pNILM by Ren et al. [28] integrated deep neural networks with encryption for privacy throughout the learning process. Alghamdi et al. [29] proposed Fed-SAD, a secure aggregation framework to safeguard forecasting models from poisoning attacks. While [27-29] papers offer useful insights into applying federated learning for privacy-preserving energy forecasting, their findings are limited by small controlled datasets, ideal communication assumptions, and minimal adversarial modeling, leaving scalability, real-world robustness, and edge-device feasibility insufficiently validated.

Cheng et al. [30] proposed a horizontally distributed FL approach that enables private edge cloud energy disaggregation. Additionally, Wang et al. [31] incorporated GRU and ensemble learning within FL to detect electricity theft, reinforcing the potential of hybrid learning structures. Liu et al. [32] further introduced a quantum enhanced FL framework (QFEVAL) to assess dynamic grid threats while minimizing communication overhead. However, all three frameworks [30-32] rely on controlled datasets, ideal communication conditions, limited adversarial modeling, and no real-device validation. While leaving their scalability, robustness, and practical deployment in large, heterogeneous smart-grid environments insufficiently demonstrated.

Blockchain Incorporation in Smart Grid Privacy and Security

Over the past couple of years, several studies have been conducted over the topic of privacy concerns and data security in smart grid. Blockchain is an emerging technology [37][50] which researchers are using for security and authorization. The use of blockchain technologies in combination with FL [11][38][39][41] is achieving more attention for ensuring integrity, accountability, and privacy [49][53] in smart grid systems. In [33] researchers presented a blockchain based framework for authentication and authorization in IoT as a solution to protect data security as well as privacy in smart cities.

Yu et al. [34] proposed a dual robust federated digital twin framework with blockchain for real time grid monitoring. Zhang et al. [35] proposed FedGrid, a model that combines the FL and blockchain mechanism, for ensuring energy usage optimization in security. Goodman et al. [36] underlined the contribution of decentralized computation in the increased edge resilience and data protection data. While these studies demonstrate valuable advancements in applying FL, blockchain, and digital-twin technologies to enhance privacy, forecasting accuracy, and coordination in smart-grid systems, their reliance on simplified datasets, idealized network conditions, and limited adversarial modeling leaves scalability and cyber-resilience in real heterogeneous deployments insufficiently validated.

Together, these studies offer foundational insights for the design and development of the proposed PPS framework, combining privacy preserving computation, distributed intelligence, and secure blockchain integration.

Table 1 presents a structured analysis of existing literature, categorized into seven research areas aligned with the architectural layers of the proposed BeFL framework. The studies cover key aspects such as privacy preserving data collection at the edge, federated load forecasting, adversarial threat mitigation, and blockchain-enabled integrity and billing.

Table 1 shows the significant contributions that can be found in the surveyed literature as it relates to federated energy prediction, blockchain enabled integrity and smart metering. Nevertheless, several critical limitations are still pervasive. Foremost, there is a lack of end to end comprehensive privacy enforcement with limited implementation of differential privacy on the client or of secure aggregation protocols for federated updates. Moreover, mechanisms for protecting identity through multi-layer hashing, role based access control, and an adaptive trust

based client selection are largely underexplored. A few studies are referring to update level tamper evidence or to enable dynamic participation under an adversarial environment.

In order to mitigate these weaknesses, the proposed BeFL framework adopts a multilayer architecture, which combines federated learning, differential privacy, blockchain based logging and secure smart contract validation. Unlike previous models, it provides update level confidentiality, hash-based identifier separation, and dynamic, trust aware client selection for secure aggregation. Additionally, it has a dual layer blockchain architecture (PrivateLayer and ProtectedLayer) to prevent re-identification, and allows fine grained access control using smart contracts, which alleviates the major privacy, security, and scalability limitations identified in previous studies.

Concern # 8: Consider expanding Table 1 to include the limitations of the reviewed works.

Authors Response: We thank the reviewer for their valuable feedback. We have now incorporated Limitation/Research Gaps in Table 1. Kindly find the updated table below:

Table 1. Critical Summary of Research Areas and Identified Gaps Aligned with the Proposed BeFL Framework

Thematic Area of Research	Core Contributions and Technical Focus	Corresponding Layer in Proposed BeFL Framework	Representative Studies	Limitations / Research Gaps
Edge Intelligence & Privacy-Preserving Data Collection	Utilizes on-device FL and smart meters (NILM) to enable distributed energy monitoring and privacy-preserving computation	Edge Device Layer, Secure Smart Meters, NILM Modules	[1], [2], [4], [8], [51]	1. Limited use of differential privacy.<br>2. No blockchain-based audit trail for data sharing.<br>3. Static client participation; lacks trust-based selection
Federated Learning for Load Forecasting and Disaggregation	Implements FL for decentralized load forecasting and appliance-level disaggregation.	FL Model Training Layer, Forecasting Engine	[1], [3], [4], [9], [50]	1. Global model parameters often exposed.<br>2. Absence of secure aggregation or DP-FedAvg<br>3. No tamper-evident model update logging
Security Against Adversarial Attacks in Federated Systems	Proposes defences against data poisoning, inference and Trojan attacks in FL systems.	Threat Detection Unit, Secure Aggregation Mechanism	[5], [6], [7], [9]	1. Focused on detection rather than prevention<br>2. No blockchain-backed verification<br>3. Lacks identity and access control layers
Blockchain Integration for Trust and Integrity	Combines blockchain and FL to record updates, ensure data provenance and decentralized trust.	Blockchain Ledger Layer, Integrity Verification Unit	[10], [11], [15], [49], [52], [53]	1. Replicates model/state on-chain → privacy leakage<br>2. No dual-layer (CID/HN) identity separation<br>3. Absence of differential privacy mechanisms
Smart Contract-Based Billing and Consent Mechanisms	Uses smart contracts for automated billing, energy trading, and user consent.	Blockchain Billing Layer, Consumer Authentication and Access Control	[11], [15], [16], [17], [48]	1. Consent and billing models not privacy-hardened<br>2. Lack of encrypted identifier mapping<br>3. No cross-layer audit or DP integration
Hybrid Edge–Cloud Coordination & Model Personalization	Explores hybrid FL for edge–cloud orchestration and personalized model updates.	Edge–Cloud Coordinator Layer, Personalized FL Module	[4], [10], [16], [47], [54]	1. Insufficient handling of heterogeneous clients<br>2. Missing secure aggregation across layers<br>3. No dynamic participation control
Next-Generation Grid: Quantum FL, Digital Twins, Grid 2.0 Vision	Presents futuristic directions such as quantum FL and federated digital twins for smart grids.	Future Expansion Layer, Privacy-by-Design and Scalable Intelligence	[10], [11], [16]	1. Conceptual; lacks implementation evidence<br>2. No concrete privacy or identity framework<br>3. No evaluation of resilience under adversarial nodes

Concern # 9: Immediately after Table 1, add a paragraph to summarize the identified research gaps. Thereafter, explain how the developed framework helps bridge these gaps.

Authors Response: We thank the reviewer for the valuable feedback. We have updated the manuscript to include a discussion under Table 1 that summarizes the limitation of research works and also how the proposed framework helps bridge these gaps. The updated manuscript is as follows:

Table 1 shows the significant contributions that can be found in the surveyed literature as it relates to federated energy prediction, blockchain-enabled integrity and smart metering. Nevertheless, several critical limitations are still pervasive. Foremost, there is a lack of end-to-end comprehensive privacy enforcement with limited implementation of differential privacy on the client or of secure aggregation protocols for federated updates. Moreover, mechanisms for protecting identity through multi-layer hashing, role-based access control, and an adaptive trust-based client selection are largely underexplored. A few studies are referring to update level tamper evidence or to enable dynamic participation under an adversarial environment.

In order to mitigate these weaknesses, the proposed BeFL framework adopts a multilayer architecture, which combines federated learning, differential privacy, blockchain-based logging and secure smart contract validation. Unlike previous models, it provides update-level confidentiality, hash-based identifier separation, and dynamic, trust-aware client selection for secure aggregation. Additionally, it has a dual-layer blockchain architecture (PrivateLayer and ProtectedLayer) to prevent re-identification, and allows fine-grained access control using smart contracts, which alleviates the major privacy, security, and scalability limitations identified in previous studies.

Concern # 10: To enhance readability, add a table of all notations/symbols used throughout this paper.

Authors Response: We thank the reviewer for the valuable feedback. We have updated the manuscript and added 2 tables below the Introduction section that detail all the different acronyms as well as the different scientific notations used in the manuscript. The tables are as follows:

Table 1. List of Acronyms

Acronyms	Description
AI	Artificial Intelligence
BeFL	Blockchain enabled Federated Learning framework
B-FRL	Blockchain-Federated Reinforcement Learning
CCPA	California Consumer Privacy Act
CID	Client ID
DP	Differential Privacy
FL	Federated Learning
GAN	Generative Adversarial Network
GDPR	General Data Protection Regulation
HGB	HistGradient Boosting
HIPAA	Health Insurance Portability and Accountability Act
HN	House Number
IID	Independent and Identically Distributed
IoT	Internet of Things
KNN	K-Nearest Neighbor
LR	Linear Regression
MAE	Mean Absolute Error
MAPE	Mean Absolute Percentage Error
ML	Machine Learning
MLP	Multi-Layer Perceptron
MSE	Mean Squared Error
PAFLM	Privacy-Preserving Asynchronous FL Mechanism
PRECON	Pakistan Residential Electricity CONsumption
QFEVAL	Quantum Federated Ensembled Variational Adaptive Learning
R^2	Coefficient of Determination
RF	Random Forest
RPC	Remote Procedure Call
SG	Smart Grid
SVR	Support Vector Regression
UUID	Universally Unique Identifier

Table 2. List of Scientific notations

Notations	Meaning
H	Set of Houses
h_k	k^{th} element in H
k	Single Household
D_k	Household Usage Data
m_k	Number of rows (reading) in D_k
n	Number of columns (features) in D_k
D'_k	Dataset after addition of CID and HN
CID_k	CID of kth household
HN_k	HN of kth household
D_{agg}	Aggregated Dataset
K	Household Datasets
$\bigcup_{k=1}^{K} D'_k$	Vertical Concatenation of Ks
M	Total number of rows for all Ks
$n+2$	Total number of features
$\mathbb{R}^{M \times (n+2)}$	Dimensions of the aggregated D_{agg}
CID_i	Original value of CID for i^{th} data entry
HN_i	Original value of HN for i^{th} data entry
$\mathcal{H}(\cdot)$	Secure Cryptographic Hash Function
$\widehat{CID_i}$	Hashed CID
$\widehat{HN_i}$	Hashed HN
s_k	Secure Random Salt
$\|$	Concatenation of s_k to CID and HN
$\mathcal{CH}_k$	Salted Hashed CID
$\mathcal{HH}_k$	Salted Hashed HN
S	l_2-sensitivity bound
θ_k	Local model Weights or parameter
ε	Privacy Budget
δ	Failure Probability
σ	Noise Level
N	Total number of FL clients
θ^{agg}	Global Model
$\tilde{\theta}^{\text{agg}}$	Noise Injected Global Model

Concern # 11: Algorithm 1 should be well described within text. In addition, it should have well defined input (s) and output (s). The same applies to all figures within this paper.

Authors Response: We are grateful for the reviewer for the valuable input. We have modified the manuscript and added a more comprehensive discussion on Algorithm 1. We have also provided the input and output in Algorithm 1. The updated manuscript is as follows:

The step-by-step implementation of the proposed framework is described in Algorithm 1 that combines the blockchain with the federated learning and central differential privacy. The procedure starts with the local processing of the distributed dataset, during which unique client

and household identifiers (Client ID, House Number) are generated and are cryptographically hashed with the assistance of salted SHA-256, thereby ensuring anonymity and integrity. These hashed identifiers are then stored securely on the Ethereum blockchain in two dedicated smart contracts, thereby ensuring verifiability and unlinkability. These hashed records are then aggregated and distributed to federated clients, where local training occurs without exposing raw data. From each client individual training, local parameters (θ_k) are calculated and sent to the central server. The central server then aggregates all the client-side parameters into a global model (θ^{agg}). The global model is then introduced to noise that comes from a Gaussian distribution to enforce (ϵ, δ) central DP. This injection of noise results in a final global model ($\theta^{\widetilde{agg}}$)

Concern # 12: In the Experimental Setup section, give rationale for the selected tools/values/settings/libraries

Authors Response: We thank the reviewer for their valuable feedback. We have updated the manuscript and added rationale for all the tools and libraries in the Experimental setup section. The updated manuscript is provided as follows:

Data Pre-processing and Aggregation

The first layer of the proposed framework consisted of obtaining the PRECON dataset which initially involved the inclusion of 42 (.csv) files with each file corresponding to a single household data. After obtaining the dataset, clusters were made with each cluster involving 4-5 household (.csv) files. The next step after this was the generation of unique identifiers in the form of ClientID and HouseNumbers using UUID-based randomization for each dataset file. The UUID-based randomization was done using the UUIDv4 library as this ensured the generation of identifiers that are random as well as collision-free while also supporting secure indexing, pseudonymity and integrity of client records. These identifiers were then added to all the dataset files with each dataset having a unique ClientID and HouseNumber as shown in \ref{fig015}. The final step is the aggregation of all the clustered dataset files into a single aggregated file. The overall handling of the dataset files as well as the aggregation was done using the Pandas library as it is extremely effective in terms of dataset loading, pre-processing and tabular manipulation.

Data Encryption and Blockchain Layer

Once the aggregated dataset from the previous layer is received in the blockchain layer, the ClientID and HouseNumber identifiers are isolated from the dataset and encrypted using salted hash using the SHA256 hash function. The use of salted hash was done using the hashlib library as it enables secure anonymization of the identifiers.

During the hashing of the identifiers, a local blockchain environment is deployed using Ganache, which is basically a local Ethereum test network that provides deterministic accounts, private and public keys and a high-speed execution environment. The framework is connected to the local chain using a Remote Procedure Call (RPC) endpoint which in turn could be accessed through a Web3 library. Once the local blockchain was created, two solidity smart contracts (PrivateLayer, ProtectedLayer) were compiled and deployed using the Truffle toolchain. The purpose of each of the smart contracts was to store the salted hashed identifiers.

Cognitive Layer

The final layer is the cognitive layer which receives the encrypted aggregate datasets at a Federated server. At this point, the dataset is divided into various subsets to cater each of the federated clients, initially set to 5 clients for the simulated environment. Once each client receives a subset of the dataset, the columns of ClientID, HouseNumber and Date_Time is removed from each dataset. The target variable is then set to Usage_kW which is then used for supervised load forecasting tasks. Different ML models, such as Random Forest (RF), XGBoost, HistGradient Boosting (HGB), Multi-layer Perceptron (MLP), Support Vector Regression (SVR), Ridge Regression, Linear Regression (LR), and K-Nearest Neighbor (KNN), are then utilized to train the received dataset. These ML models are loaded using the Scikit-Learn library which is also utilized for the different performance metrics considered for this simulation.

In order to simulate an FL based environment, the Flower framework is utilized which enables communication between multiple FL clients and a central server. The reason for the selection of the Flower framework is because unlike other FL based frameworks, Flower provides simulation of multiple clients, secure aggregation of model updates, customization of strategies (e.g. FedAvg, FedAdam, FedProx etc.), integration with existing PyTorch/Tensorflow models. Once a FL client has trained their respective dataset, the Flower framework ensures that only the

weights/parameters from each training are transferred to the central server using the FedAvg algorithm. Once different parameters are received at the central server, they are aggregated together to become a global model. After the aggregation is complete, zero-mean Gaussian noise is injected into the global model using NumPy library. This injection of noise ensures the implementation of the central DP mechanism. This noise injection is done after every communication round. Once the noise has been inserted, the noisy global model is then transmitted back to the FL clients for another communication round.

Concern # 13: Implementation details of the Federated Learning have not been adequately described. The same can be said of Data Pre-processing.

Authors Response: We thank the reviewers for their valuable feedback. We have updated the manuscript accordingly and provided more details on data-pre-processing on every layer as well as for the implementation details of Federated Learning. The updated changes for each layer are as follows:

Data Pre-processing and Aggregation

The first layer of the proposed framework consisted of obtaining the PRECON dataset which initially involved the inclusion of 42 (.csv) files with each file corresponding to a single household data. After obtaining the dataset, clusters were made with each cluster involving 4-5 household (.csv) files. The next step after this was the generation of unique identifiers in the form of ClientID and HouseNumbers using UUID-based randomization for each dataset file. The UUID-based randomization was done using the UUIDv4 library as this ensured the generation of identifiers that are random as well as collision-free while also supporting secure indexing, pseudonymity and integrity of client records. These identifiers were then added to all the dataset files with each dataset having a unique ClientID and HouseNumber as shown in \ref{fig015}. The final step is the aggregation of all the clustered dataset files into a single aggregated file. The overall handling of the dataset files as well as the aggregation was done using the Pandas library as it is extremely effective in terms of dataset loading, pre-processing and tabular manipulation.

Data Encryption and Blockchain Layer

Once the aggregated dataset from the previous layer is received in the blockchain layer, the ClientID and HouseNumber identifiers are isolated from the dataset and encrypted using salted hash using the SHA256 hash function. The use of salted hash was done using the hashlib library as it enables secure anonymization of the identifiers.

During the hashing of the identifiers, a local blockchain environment is deployed using Ganache, which is basically a local Ethereum test network that provides deterministic accounts, private and public keys and a high-speed execution environment. The framework is connected to the local chain using a Remote Procedure Call (RPC) endpoint which in turn could be accessed through a Web3 library. Once the local blockchain was created, two solidity smart contracts (PrivateLayer, ProtectedLayer) were compiled and deployed using the Truffle toolchain. The purpose of each of the smart contracts was to store the salted hashed identifiers.

Cognitive Layer

The final layer is the cognitive layer which receives the encrypted aggregate datasets at a Federated server. At this point, the dataset is divided into various subsets to cater each of the federated clients, initially set to 5 clients for the simulated environment. Once each client receives a subset of the dataset, the columns of ClientID, HouseNumber and Date_Time is removed from each dataset. The target variable is then set to Usage_kW which is then used for supervised load forecasting tasks. Different ML models, such as Random Forest (RF), XGBoost, HistGradient Boosting (HGB), Multi-layer Perceptron (MLP), Support Vector Regression (SVR), Ridge Regression, Linear Regression (LR), and K-Nearest Neighbor (KNN), are then utilized to train the received dataset. These ML models are loaded using the Scikit-Learn library which is also utilized for the different performance metrics considered for this simulation.

In order to simulate an FL based environment, the Flower framework is utilized which enables communication between multiple FL clients and a central server. The reason for the selection of the Flower framework is because unlike other FL based frameworks, Flower provides simulation of multiple clients, secure aggregation of model updates, customization of strategies (e.g. FedAvg, FedAdam, FedProx etc.), integration with existing PyTorch/Tensorflow models. Once a FL client has trained their respective dataset, the Flower framework ensures that only the weights/parameters from each training are transferred to the central server using the FedAvg algorithm. Once different parameters are received at the central server, they are aggregated

together to become a global model. After the aggregation is complete, zero-mean Gaussian noise is injected into the global model using NumPy library. This injection of noise ensures the implementation of the central DP mechanism. This noise injection is done after every communication round. Once the noise has been inserted, the noisy global model is then transmitted back to the FL clients for another communication round.

Concern # 14: Give the mathematical formulations of the deployed metrics, such as MAPE, MAE & MSE.

Authors Response: We thank the reviewers for their valuable feedback. We have updated the manuscript to include all the mathematical formulations of the deployed metrics. The updated manuscript is as follows:

Performance Evaluation Metrics

The predictive accuracy of the proposed framework is evaluated by the following statistical metrics:

- Mean Absolute Error (MAE)

$$\text{MAE} = \frac{1}{n}\sum_{i=1}^{n}|y_i - \hat{y}_i| \tag{12}$$

- Mean Squared Error (MSE)

$$\text{MSE} = \frac{1}{n}\sum_{i=1}^{n}(y_i - \hat{y}_i)^2 \tag{13}$$

- Mean Absolute Percentage Error (MAPE)

$$\text{MAPE} = \frac{100\%}{n}\sum_{i=1}^{n}\left|\frac{y_i - \hat{y}_i}{y_i}\right| \tag{14}$$

- Coefficient of Determination (R^2)

$$R^2 = 1 - \frac{\sum_{i=1}^{n}(y_i - \hat{y}_i)^2}{\sum_{i=1}^{n}(y_i - \bar{y})^2}, \quad \text{where} \quad \bar{y} = \frac{1}{n}\sum_{i=1}^{n} y_i \tag{15}$$

Concern # 15: The clarity of all figures must be improved.

Authors Response: We thank the reviewer for the valuable comments. We have updated all of the Figures present in the manuscript.

Concern # 16: Describe how the identified limitations of the proposed framework can be addressed.

Authors Response: We thank the reviewer for the valuable feedback. We have updated the Limitation and Future Works section in the manuscript to provide a more detailed description of all the limitations presented in the proposed framework. The updated manuscript is as follows:

Limitations and Future Work

Although the proposed federated learning framework with blockchain integration provides significant capabilities with respect to preserving privacy and providing exact analytics with respect to energy usage, several limitations became apparent in the course of implementation. Experimental validation was done as IID conditions, but realistic smart grid data mostly contain non-IID characteristics with respect to variable consuming patterns (which may have adverse effect on the federated model with regard to convergence and generalization of the model).

The blockchain layer, although providing secure and unalterable logging, adds latency and increases gas costs. While these metrics stayed within acceptable limits during simulations, scaling up to larger implementations could intensify these issues. Adjusting differential privacy parameters indicated a trade-off between privacy preservation and model accuracy which showed a need for future research into adaptive privacy schemes. Moreover, anomalous residuals seen for specific clients even more strongly illustrated the influence of uneven data distributions that indicated the need for more advanced client-side training adaptations.

Future work should investigate methodologies to provide better support for non-IID data to increase the scalability and resilience in heterogeneous data distributions. This includes adopting

resource aware model training on edge devices and looking into efficient blockchain alternatives. Additionally, the integration of advanced privacy preservation techniques, such as homomorphic encryption or secure aggregation, as well as the extension of the framework to handle deep learning models for time series forecasting is something that will certainly increase its applicability to practical smart grid scenarios.

Reviewer #2:

Concern # 1: Can you provide more details on the dual-layer blockchain architecture and its implementation?

Authors Response: We thank the reviewer for their valuable feedback. We have updated the manuscript to provide more detail on the dual-layer blockchain architecture and its implementation. The updated manuscript is as follows:

Data Encryption and Blockchain Layer

Once the aggregated dataset from the previous layer is received in the blockchain layer, the ClientID and HouseNumber identifiers are isolated from the dataset and encrypted using salted hash using the SHA256 hash function. The use of salted hash was done using the hashlib library as it enables secure anonymization of the identifiers.

During the hashing of the identifiers, a local blockchain environment is deployed using Ganache, which is basically a local Ethereum test network that provides deterministic accounts, private and public keys and a high-speed execution environment. The framework is connected to the local chain using a Remote Procedure Call (RPC) endpoint which in turn could be accessed through a Web3 library. Once the local blockchain was created, two solidity smart contracts (PrivateLayer, ProtectedLayer) were compiled and deployed using the Truffle toolchain. The purpose of each of the smart contracts was to store the salted hashed identifiers.

1. Private Layer Smart Contract

The PrivateLayer smart contract is used to maintain an on-chain registry of the salted HouseNumber. Whenever a new hash is submitted using the PrivateLayer contract, the contract checks whether the HN has already been stored or not. If the HN has not been stored, a new hash

is mapped, and an event is emitted. A read-only verification function also allows external components to verify whether a particular HouseNumber hash has been recorded previously without the revealing of the original house number. A detailed working of the PrivateLayer smart contract is shown in Algorithm 2.

Algorithm 2 PrivateLayer: House Hash Registration and Verification

```
State: submittedHouseHashes : mapping(string → bool)
Event: HouseHashSubmitted(houseHash)
function SUBMITHOUSENUMBER(houseHash)
    Input: houseHash (salted SHA-256 hash of the house identifier)
    Output: None (writes hash to blockchain state)
Require: houseHash ≠ ""
Require: submittedHouseHashes[houseHash] = false
    submittedHouseHashes[houseHash] ← true
    emit HouseHashSubmitted(houseHash)
    return
end function
function VERIFYHOUSEHASH(houseHash)
    Input: houseHash (salted SHA-256 hash)
    Output: exists ∈ {true, false}
    return submittedHouseHashes[houseHash]
end function
```

2. Protected Layer Smart Contract

The ProtectedLayer smart contract is used for the storing of the salted ClientID. Through this contract, each new ClientID hash is validated for uniqueness to prevent duplication or replay. Once a new hashed ClientID is registered successfully, similar to the PrivateLayer, a mapping is recorded, and an event is emitted. A verification function applies a constant time check to verify whether a ClientID hash already exists on-chain or not. This function enables identity traceability while keeping the raw ClientID off-chain. Algorithm 3 provides a descriptive working of the ProtectedLayer smart contract.

Algorithm 3 ProtectedLayer: Client Hash Registration and Verification

State: submittedClientHashes : mapping(string $\rightarrow$ bool)
Event: ClientHashSubmitted(clientHash)
function SUBMITCLIENTID(clientHash)
    Input: clientHash (salted SHA-256 hash of the client identifier)
    Output: None (writes hash to blockchain state)
Require: clientHash $\neq$ ""
Require: submittedClientHashes[clientHash] = false
    submittedClientHashes[clientHash] $\leftarrow$ true
    emit ClientHashSubmitted(clientHash)
    return
end function
function VERIFYCLIENTHASH(clientHash)
    Input: clientHash (salted SHA-256 hash)
    Output: exists $\in$ {true, false}
    return submittedClientHashes[clientHash]
end function

After the transaction submission of both the ClientID and HouseNumber to their respective smart contracts finishes, the blockchain provides a successful receipt that provides that the transaction has been mined at the end of the blockchain pipeline. Also provided is the gas utilized, execution time as well as latency that would be used for performance analysis later. Once the hashed identifiers are received after passing through the blockchain, these hashed identifiers are placed in the aggregated dataset, replacing the raw identifiers. This results in an encrypted dataset with hashed identifiers and the original energy features.

Concern # 2: How does the framework handle data heterogeneity and variability in smart grid environments?

Authors Response: We thank the reviewer for their valuable feedback. We have incorporated a new subsection in the Experimentation section, titled "Discussion", where we have incorporated the changes requested.

Impact of Data Heterogeneity and Bias

A significant feature of real-world smart grid scenarios is that there is data heterogeneity, i.e. each household or smart meter can have distinct consumption patterns, usage behaviors and

machine-level variations. Although the existing implementation of the framework uses the IID partitioning of the PRECON dataset to set a baseline performance and test the core functionality, the federated learning design would naturally provide decentralized learning using heterogeneous data. The use of the Federated Averaging (FedAvg) algorithm mitigates the influence of local biases by proportionally weighting client updates based on dataset size, promoting generalizable learning over overfitting to localized anomalies. In addition, the incorporation of central differential privacy during the aggregation phase introduces Gaussian noise, which statistically smooths extreme or skewed updates, offering additional robustness against client-specific biases. Moreover, the implementation of central differential privacy at the aggregation step also brings statistical smoothing of Gaussian noise that weakens the effect of extreme updates or skewed updates of individual clients. Even though the heterogeneity of the data was not directly represented with the help of the model in this paper, the next steps will be to simulate non-IID data and add personalization strategies or clients selection mechanisms to the framework in order to ensure the enhancement of the resilience to the variability of the real smart grid.

Concern # 3: Can you discuss the trade-off between privacy and accuracy in the proposed framework?

Authors Response: We thank the reviewer for their valuable feedback. We have updated the manuscript and added the following in the Discussion section:

Critical Trade-offs

The proposed BeFL framework has aimed to create a balance between maintaining client privacy and preservation of model accuracy by incorporating central DP. This is done by injecting Gaussian noise to the aggregated model at the cognitive layer in order to hide the identification of all the participating clients. Through this approach, the risk of data leakage is mitigated while also reducing the risk of reconstruction attacks. However, with the injection of noise, a loss of model performance was observed. In order to counter this issue, the BeFL framework utilizes the possibility of adjustment of noise levels according to the sensitivity of the dataset distribution and aggregation function. Another approach utilized was the clipping of local model updates to a predefined norm which limited the variance before the injection of Gaussian noise. These approaches ensured that the BeFL performed relatively better as seen in MSE and MAE,

discussed in previous section, while still preserving the privacy of clients and adhering to regulatory compliance.

Concern # 4: How does the framework ensure regulatory compliance with standards such as GDPR and CCPA?

Authors Response: We thank the reviewer for the valuable feedback. We have updated the manuscript and incorporated regulatory compliance with our proposed framework in the Introduction Section. The updated manuscript is as follows:

The proposed Blockchain enabled Federated Learning framework (BeFL) ensures data protection standards suggested by General Data Protection Regulation (GDPR [12] and California Consumer Privacy Act (CCPA) [13]. The proposed framework BeFL ensures that raw data remains on client devices, fulfilling data minimization requirements. While unique identifiers are pseudonymized using UUID and SHA-256 with salt. These encrypted hashes are stored on an Ethereum blockchain via smart contracts, enabling immutable audit trails and accountability. The framework avoids direct transmission of personal identifiers and supports opt-in mechanisms. Thus, maintaining compliance with consent, transparency, and de-identification provisions which are mandated by GDPR and CCPA.

The proposed framework is not restricted to SGs for forecasting, but it can be used in other sectors, where data privacy and trust are of the most importance. In the medical field it can enable model training across hospitals while adhering to data protection laws like Health Insurance Portability and Accountability Act (HIPAA) [14] and GDPR [12]. In smart cities, it can be used for privacy aware mobility or environmental forecasting using edge IoT devices. In the financial sector, the framework can be applied to identify fraud or to optimize the prediction of risk without exposing client sensitive transactional data. Additionally, for the industrial IoT environments, it helps support predictive maintenance and process optimization across the distributed manufacturing units while protecting proprietary information. By maintaining data locality, input origin verifiability, and individual contribution protection, the framework provides a scalable, secure, and privacy-conscious decentralized AI solution for multi domain use.

Concern # 5: Can you provide more information on the Flower framework and its role in implementing the proposed system?

Authors Response: We thank the reviewer for the valuable feedback. We have updated the manuscript and provided a more comprehensive description on Flower Framework and its role in implementing the proposed system in the Experimental Setup section. The updated manuscript is as follows:

Cognitive Layer

The final layer is the cognitive layer which receives the encrypted aggregate datasets at a Federated server. At this point, the dataset is divided into various subsets to cater each of the federated clients, initially set to 5 clients for the simulated environment. Once each client receives a subset of the dataset, the columns of ClientID, HouseNumber and Date_Time is removed from each dataset. The target variable is then set to Usage_kW which is then used for supervised load forecasting tasks. Different ML models, such as Random Forest (RF), XGBoost, HistGradient Boosting (HGB), Multi-layer Perceptron (MLP), Support Vector Regression (SVR), Ridge Regression, Linear Regression (LR), and K-Nearest Neighbor (KNN), are then utilized to train the received dataset. These ML models are loaded using the Scikit-Learn library which is also utilized for the different performance metrics considered for this simulation.

In order to simulate an FL based environment, the Flower framework is utilized which enables communication between multiple FL clients and a central server. The reason for the selection of the Flower framework is because unlike other FL based frameworks, Flower provides simulation of multiple clients, secure aggregation of model updates, customization of strategies (e.g. FedAvg, FedAdam, FedProx etc.), integration with existing PyTorch/Tensorflow models. Once a FL client has trained their respective dataset, the Flower framework ensures that only the weights/parameters from each training are transferred to the central server using the FedAvg algorithm. Once different parameters are received at the central server, they are aggregated together to become a global model. After the aggregation is complete, zero-mean Gaussian noise is injected into the global model using NumPy library. This injection of noise ensures the implementation of the central DP mechanism. This noise injection is done after every communication round. Once the noise has been inserted, the noisy global model is then transmitted back to the FL clients for another communication round.

Concern # 6: How does the framework handle model updates and aggregation in the presence of faulty or malicious clients?

Authors Response: We thank the reviewer for their valuable comment. We have updated the manuscript to incorporate the changes in the Discussion section as follows:

Robustness and Explainability

The proposed framework strengthens both the robustness and explainability of FL by integrating multi-layer safeguards with auditable transparency mechanisms. Robustness is achieved by norm clipping, which basically bounds the magnitude of each FL client local model updates and prevents any client, either faulty or malicious, from inordinately influencing the global model, thus mitigating poisoning risks. Also, client participation is restricted to only those whose identities have been securely registered via the BeFL framework blockchain, which not only ensures authentication but also reduces the likelihood of unauthorized model submission and Sybil attacks. Furthermore, during the aggregation phase of local updates, through the employed FedAvg algorithm strategy, the updates are weighed according to the relative size of each client dataset which potentially filters out inconsistent deviant updates. These approaches ensure that the central server is able to mitigate the influence of nodes that are compromised and guarantee that the training process remains reliable.

Concern # 7: Can you discuss the potential applications of the proposed framework beyond smart grids?

Authors Response: We thank the reviewer for their valuable feedback. We have updated the manuscript and incorporated the changes in the Discussion section as follows:

Real World Implications and Applications

The proposed framework BeFL includes federated learning, identity protection with the help of blockchain, and differential privacy. Other than SG, it has significant applicability in various areas where sensitive distributed data is utilized. In healthcare, for instance, hospitals or diagnostic centers could collaboratively train predictive models on patient data without sharing

raw medical records, while blockchain ensures traceable participation and privacy preserving auditability. In finance, local records of transactions could be used to identify fraud or evaluate risk through the decentralized banking or insurance providers who protect the identities of clients. Likewise, in industrial IoT contexts, where confidentiality and integrity of data are the key concerns, the framework can support secure cooperation of distributed edge devices. The layered architecture has a modularity that allows adopting domain specific needs and makes it a flexible system to solve the privacy preserving, trustful, and decentralized machine learning in controlled industries.

Concern # 8: How does the framework ensure transparency and explainability in FL model updates?

Authors Response: We thank the reviewer for their valuable comment. We have updated the Discussion section in the manuscript to incorporate the following changes:

Robustness and Explainability

The aspects of explainability and transparency is achieved by the BeFL framework in the blockchain phase where the ClientID and House Number identifiers are first salted and then hashed. These salted and hashed identifiers are then transferred into the blockchain. Through the maintenance of each round records involving model updates, client participation, and details of aggregations, stakeholders are able to perform post-hoc analysis. From this analysis, it is possible to monitor performance fluctuations and anomalies of specific rounds or involved clients. From these methods, it is possible to ensure that the process of model training remains transparent for regulatory compliance and accountability.

Concern # 9: Can you provide more details on the Gaussian noise application and its impact on model performance?

Authors Response: We thank the reviewer for their valuable comments. We have updated the manuscript and provided more details of Gaussian noise application and its impact on model performance in the Discussion section, specifically in the Critical Trade-off section.

The proposed BeFL framework has aimed to create a balance between maintaining client privacy and preservation of model accuracy by incorporating central DP. This is done by injecting Gaussian noise to the aggregated model at the cognitive layer in order to hide the identification of all the participating clients. Through this approach, the risk of data leakage is mitigated while also reducing the risk of reconstruction attacks. However, with the injection of noise, a loss of model performance was observed. In order to counter this issue, the BeFL framework utilizes the possibility of adjustment of noise levels according to the sensitivity of the dataset distribution and aggregation function. Another approach utilized was the clipping of local model updates to a predefined norm which limited the variance before the injection of Gaussian noise. These approaches ensured that the BeFL performed relatively better as seen in MSE and MAE, discussed in previous section, while still preserving the privacy of clients and adhering to regulatory compliance.

Concern # 10: Highlighted article might be considered for related work. (Dhasarathan, C., Ramachandra, R. B., & Tripathi, D. Safeguarding User Privacy at the Edge of Fog Computing Networks in Decentralized Distributed Computing. In Swarm Intelligence (pp. 133-155). CRC Press.)

Authors Response: We thank the reviewer for the valuable feedback. We have incorporated the research work provided in the manuscript in Table 1. The reference location is [54] and is listed as follows:

54. Dhasarathan, C., Ramachandra, R. B., & Tripathi, D. Safeguarding User Privacy at the Edge of Fog Computing Networks in Decentralized Distributed Computing. In Swarm Intelligence (pp. 133-155). CRC Press.

Concern # 11: How does the framework plan to address potential biases and disparities in FL model updates and predictions?

Authors Response: We thank the reviewer for their valuable comments. We have updated the manuscript and incorporated the changes in the Discussion section as follows:

Impact of Data Heterogeneity and Bias

A significant feature of real-world smart grid scenarios is that there is data heterogeneity, i.e. each household or smart meter can have distinct consumption patterns, usage behaviors and machine-level variations. Although the existing implementation of the framework uses the IID partitioning of the PRECON dataset to set a baseline performance and test the core functionality, the federated learning design would naturally provide decentralized learning using heterogeneous data. The use of the Federated Averaging (FedAvg) algorithm mitigates the influence of local biases by proportionally weighting client updates based on dataset size, promoting generalizable learning over overfitting to localized anomalies. In addition, the incorporation of central differential privacy during the aggregation phase introduces Gaussian noise, which statistically smooths extreme or skewed updates, offering additional robustness against client-specific biases. Moreover, the implementation of central differential privacy at the aggregation step also brings statistical smoothing of Gaussian noise that weakens the effect of extreme updates or skewed updates of individual clients. Even though the heterogeneity of the data was not directly represented with the help of the model in this paper, the next steps will be to simulate non-IID data and add personalization strategies or clients selection mechanisms to the framework in order to ensure the enhancement of the resilience to the variability of the real smart grid.
